# Pattern and predictors of medicine use among households in Gondar Town, northwestern Ethiopia: a community-based medicine utilization study

**DOI:** 10.1186/s13104-017-2669-7

**Published:** 2017-07-28

**Authors:** Fitsum Sebsibe Teni, Eshetie Melese Birru, Abdrrahman Shemsu Surur, Assefa Belay, Dawit Wondimsigegn, Dessalegn Asmelashe Gelayee, Zewdneh Shewamene

**Affiliations:** 1 Department of Pharmaceutics and Social Pharmacy, School of Pharmacy, College of Health Sciences, Addis Ababa University, Addis Ababa, Ethiopia; 2 Department of Pharmacology, School of Pharmacy, College of Medicine and Health Sciences, University of Gondar, Gondar, Ethiopia; 3 Department of Pharmaceutical Chemistry, School of Pharmacy, College of Medicine and Health Sciences, University of Gondar, Gondar, Ethiopia; 4 Department of Pharmaceutics and Social Pharmacy, School of Pharmacy, College of Medicine and Health Sciences, University of Gondar, Gondar, Ethiopia

**Keywords:** Ethiopia, Gondar, Medicine use, Medicine sharing, Medicine disposal

## Abstract

**Background:**

Medicine use can be influenced by several factors. Health managers need specific information about irrational use of medicines, in order to identify opportunities to enhance rational use of medicines in their communities. This study aimed to assess the pattern and factors associated with household medicine use in Gondar town, northwestern Ethiopia.

**Methods:**

An interviewer-administered cross-sectional survey was conducted on 771 households, carried out between 5 April and 6 May 2015. The questionnaire contained items focusing on different aspects of medicine use in the households. The analysis involved descriptive summary and binary logistic regression test, which assessed association of independent variables with medicine use.

**Results:**

Of the households interviewed, 22.4% (173/771) disclosed the presence of at least one chronic disease in the family; while 49.2% reported the use of medicine in the one month prior to the study. Almost all of the households (92.6%) reported a habit of discontinuing medicines, and 17.8% disclosed a practice of sharing medicines with household members and others. Level of education, presence of health professionals, and individuals with chronic illness in the households were linked to increased likelihood of reporting medicine use. Discarding leftover medicines with garbage (56.7%) was the principal means of disposal reported by the households.

**Conclusions:**

A high proportion of reported medicine use, together with problems such as sharing with other people and leaving medicines unfinished were found among the households in the study.

## Background

Annual global spending on medicines was estimated to reach nearly 1.2 trillion United States Dollars by 2016, with the top 20 therapy areas accounting for 42% of the total, where cancer, diabetes and asthma/chronic obstructive pulmonary disease (COPD) take the lead [1]. Despite this, globally, one-third of the population lacks regular access to essential medicines. This reaches up to 50% in the poorest nations of Africa and Asia. About 50% of the medicines available are prescribed, dispensed and sold inappropriately, while half of patients do not take their medicines appropriately [2].

Different factors can be said to determine the use of medicines in the community. These include knowledge about use, the cost of medicines, regulatory systems, cultural factors, community beliefs and communication with prescribers among others [3]. These factors play out at different levels including household and community. The way medicines are used is also influenced by individual beliefs about them, which may have been shaped by members of the immediate family and those in extended family networks at household level. The community is the immediate context in which individuals and families deal with their health problems. People talk to each other about therapies, creating and reinforcing existing medicine use cultures, and they rely on local sources of medicines. In order to address the problem of irrational use of medicines, health planners and administrators require specific information on the types, extent and the reasons of irrational use of medicines [4].

Several studies on medicine utilization have been conducted globally as well as in Ethiopia, focusing mainly on health institutions [5–9]. A number of studies regarding utilization of medicines in the community have also been conducted in different countries [10–16].

In Ethiopia, medicine utilization studies among households are very scarce, restricted to self-medication and currently available medicines at home [17–20]. To the best of literature search done, no study documenting overall medicine use among households in Gondar town, was found. The aim of this study was to generate information on the pattern medicine use and related factors among households; and identify areas of improvement for enhanced rational use of medicines in Gondar town, northwestern Ethiopia.

## Methods

### Study area

The study was conducted among households in selected sub-cities of Gondar Town. It is located 750 km away from Addis Ababa, the capital city of Ethiopia. It was estimated that 224,000 people lived in Gondar town in 2014/2015 [21]. The town is divided into 24 ‘kebeles’, 12 of which are classified as sub-cities, 11 as rural ‘kebeles’ and one as a special ‘kebele’ (Gondar Town Administration. Administrative classification of ‘kebeles’ in Gondar town, 2014, unpublished). ‘Kebeles’ are the smallest administrative divisions in Ethiopia. The town has one public referral/teaching hospital and 7 health centers. The number of private health facilities in the town is 50, including private clinics and one general hospital. In addition, there are 53 medicine retail outlets including pharmacies and drug stores (Gondar Town Health Bureau. Report on the number of medicines retail outlets in Gondar town 2014, unpublished) [22].

### Study design

A community-based cross-sectional study of medicine use among households in the town, based on interviewer-administered questionnaires, was conducted.

### Sampling

Of the 24 ‘kebeles’ in Gondar town, 12 are located in the urban area of the town. Due to financial limitations the study was restricted to households found in these sub-cities. The number of households selected to be included, was determined through single proportion formula as follows: $\left[ {N = \frac{{\left( {z_{1 - \propto /2} } \right)^{2} \times p\left( {1 - p} \right)}}{{\delta^{2} }}} \right]$. The formula was used because it provides the appropriate sample size to measure proportion of medicine use in the population (as it is a single proportion). The calculation considered proportion ($p$) of households, where medicine(s) has/have been used in the previous 1 month to be 50%, to get the maximum possible sample. The margin of error allowed ($\delta$) was set at 5% and level of significance ($z_{1 - \propto /2}$) was taken to be 1.96 at 95% confidence interval (CI) [23]. In addition, a design effect of 2 was used in the sample size calculation to account for the two-stage sampling. A 5% contingency was also added, making the final sample size 809 households.

**Fig. 1:**
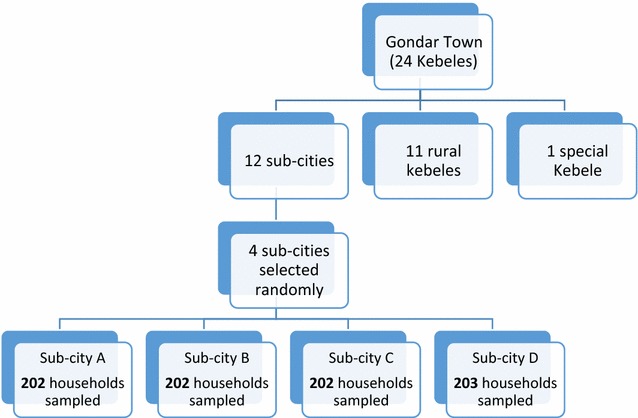
A flow chart of the sampling procedure followed in selecting households included in the study, Gondar Town, 2015

In selecting the households, a two-stage sampling was followed. First, four sub-cities were randomly selected from the 12 sub-cities of Gondar Town. This considered the representativeness of the sampled sub-cities, which accounted for one-third of the total sub-cities, and feasibility in terms of data collection. In the second stage, the selection of individual households involved equally dividing the total sample to the four sub-cities. This was followed by randomly selecting the households from each of the four sub-cities (Fig. 1).

### Data collection instrument and process

Four well trained interviewers, who held diploma level of qualification in pharmacy, conducted data collection from 5 April to 6 May 2015. The instrument used was prepared based on previous studies and guidelines [4, 13]. It was first prepared in English and then translated into Amharic. This version was back translated to English to make sure it retained its meaning during translation.

The instrument comprised two parts; the first part focused on the socio-demographic characteristics of respondents and their families. In the second part, questions concerning the details of medicine use in the family were included. A pretest was conducted among 50 households which were excluded from the final analysis. On the basis of the findings from the pretest, appropriate modifications were instituted to the instrument.

Adults (aged 18 years or older) available in the household during the data collection were used as household’s representative. In the event of unavailability of adults, a household was replaced by the one next to it after two successive visits.

### Data analysis

The data collected was entered into Epidata version 3.1 (Epidata Association, Odense, Denmark) and exported to SPSS version 21 (IBM Corp. IBM SPSS Statistics for Windows, Armonk, NY: IBM Corp. Released 2012) for analysis. Data on socio-demographic profiles and medicine use were summarized using descriptive statistics. Binary logistic regression was performed to identify predictors of medicine use. In the statistical tests conducted, the significance level (p value) was set to be <0.05 at 95% CI.

## Results

### Socio-demographic characteristics of respondents

Surveys of 771 households out of 809 were complete, and included in the final analysis, making the response rate 95.3%. The majority of respondents were female; and more than two-fifths were in the age group of 18–29 years. Housewives (40.3%) constituted the highest proportion in terms of occupational status (Table 1).

**Table 1:** Socio-demographic characteristics of respondents and their households, Gondar Town, 2015 (n = 771)

Variable	Number	Percent
Sex		
Male	183	23.7
Female	588	76.3
Age (years)		
18–29	315	40.9
30–39	177	23.0
40–49	127	16.5
50–59	68	8.8
60+	84	10.9
Religion		
Orthodox christianity	565	73.3
Islam	159	20.6
Protestantism	31	4.0
Others^a^	16	2.1
Ethnicity		
Amhara	696	90.3
Tigre	47	6.1
Others^b^	28	3.6
Educational status		
Can’t read or write	162	21.0
Can read and write	87	11.3
Primary education	98	12.7
Secondary education	249	32.3
College/university education	175	22.7
Occupation		
Not working/unemployed	36	4.7
Housewife	311	40.3
Student	49	6.4
Retiree	30	3.9
Government employee	106	13.7
Private company employee	95	12.3
Merchant	141	18.3
Farmer	3	0.4
Highest education level in family		
Reading and writing	26	3.4
Primary education	94	12.2
Secondary education	234	30.4
College/university education	417	54.1
Family’s monthly income (USD)		
Up to 50	164	21.3
51–100	137	17.8
101–150	110	14.3
151–200	76	9.9
200–250	80	10.4
>250	56	7.3
Not disclosed	148	19.2

^a^Judaism, Catholicism

^b^Qimant, Oromo

More than half of the households, (54.1%) had family members who completed or were attending a college/university education. Regarding monthly income, 39.1% of the households earned up to 100 USD (Table 1).

### Medicine use in the households

**Fig. 2:**
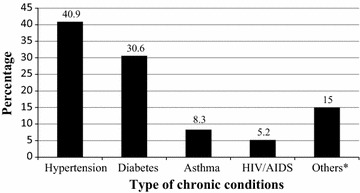
Percentage distribution of individual chronic illnesses reported among households, Gondar Town, 2015 (n = 193). *Psychiatric illness, chronic kidney disease, heart failure, cancer

Medicine use by a family member in the one month prior to the study was reported by about half (49.2%) of the households. Nearly a quarter (22.4%) also reported the presence of at least one member with a chronic illness. Of the reported illnesses, hypertension and diabetes were the most common. A considerable proportion (14.0%) of the respondents expressed the presence of a health professional in the family (Table 2; Fig. 2).

**Table 2:** Health status and medicine utilization of respondents’ households, Gondar Town, 2015 (n = 771)

Variable	Yes	
	Number	Percent
Chronic illness in the household	173	22.4
Health professional in household	108	14.0
Medication use in the previous month	379	49.2
Practice of sharing medicines	137	17.8
Leaving medicines unfinished	714	92.6
Presence of medicines at home currently	341	44.2

The practice of sharing medicines, with family members or friends/neighbors, was reported by 17.8% of the households. In addition, leaving medicines unfinished was disclosed by almost all, (92.6%) of the households. Nearly half (44.2%) of the respondents reported the presence of at least one medicine at the time of data collection (Table 2).

**Fig. 3:**
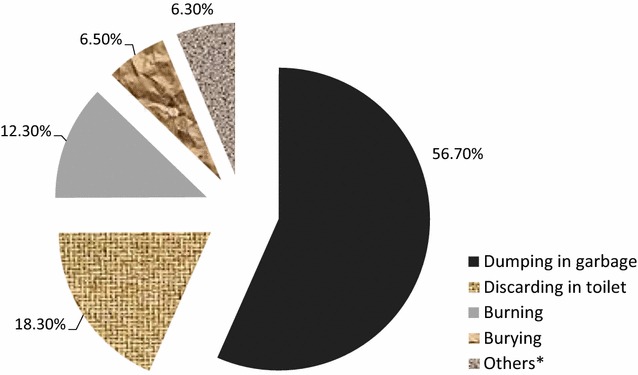
Modes of discarding unused medicines from households, Gondar towns, 2015. *Throwing away, putting in streams

The majority of the households, (80.3%; 619/771) reported a practice of discarding medicines which are expired or no longer used. Of these households, more than half (56.7%) discarded the medicines with garbage (Fig. 3).

### Predictors of medicine use

A binary logistic regression test was conducted to assess the association of socio-demographic and other variables with medicine use in the households. On the basis of the test, presence of a family member with a secondary level education (Adjusted odds ratio (AOR) = 2.667, 95% CI 1.008–7.061) was associated with medicine use in a statistically significant manner. Although college education, (AOR = 3.212, 95% CI 1.322–7.803), was also associated with medicine use in the bivariate analysis, it lost its statistical significance in the multivariate analysis (Table 3).

**Table 3:** Binary logistic regression test for predictors of medicines use in the past month among households, Gondar Town, 2015

Variable	Total number of households	Medicines utilization in the last 1 month		OR (95% CI)	
		Number	Percent	Unadjusted	Adjusted^b^
Highest level of education in the family					
Reading and writing^a^	26	7	26.9		
Primary education	94	31	33.0	1.34 (0.51–3.51)	1.45 (0.52–4.09)
Secondary education	234	115	49.1	2.62 (1.06–6.48)	2.67 (1.01–7.06)
College/university education	417	226	54.2	3.21 (1.32–7.80)	2.12 (0.79–5.68)
Presence of health professional in household					
Yes	663	307	46.3	2.32 (1.51–3.56)*	1.81 (1.10–2.96)*
No^a^	108	72	66.7		
Chronic illness in household					
Yes	598	239	40.0	6.37 (4.22–9.63)*	6.34 (4.15–9.67)*
No^a^	173	140	80.9		
Household monthly income (USD)					
<50^a^	164	67	40.9		
51–100	137	67	48.9	1.39 (0.88–2.19)	1.14 (0.69–1.88)
101–150	110	64	58.2	2.01 (1.23–3.29)*	1.52 (0.88–2.65)
151–200	76	39	51.3	1.53 (0.88–2.64)	1.17 (0.26–2.20)
201–250	80	50	62.5	2.41 (1.39–4.18)*	1.60 (0.84–3.06)
>250	56	34	60.7	2.24 (1.20–4.16)*	1.87 (0.93–3.77)
Not disclosed	148	58	39.2	0.93 (0.59–1.47)	0.85 (0.52–1.38)

* p < 0.05

^a^Reference category

^b^Adjusted for education level, presence of health professional in the household, presence of a person with chronic conditions and monthly household income

The presence of an individual with a chronic illness, (AOR = 6.336, 95% CI 4.150–9.672), also showed a statistically significant association with medicine use in the household. This indicated a more than six times likelihood of reporting medicine use in such a household (Table 3).

Households where health professionals lived were nearly two times more likely, (AOR = 1.807, 95% CI 1.104–2.959), to report medicine use than households without professionals. However, the monthly income of the households showed no statistically significant association with medicine use (Table 3).

## Discussion

This study assessed the extent and predictors of medicine use among households in Gondar town. It showed that nearly half of the households reported medicine use in the past one month. Leaving medicines unfinished was reported as a very common practice by the households. In addition, a considerable level of sharing medicines was found with more than one in six households reportedly engaged in it. As to predictors of medicine use, secondary school education, presence of a person with chronic illness and a health professional were all found to increase likelihood of reporting medicine use in the households.

Looking at the details of the findings, more than a fifth were found to have at least one member with chronic illness. This was consistent with past studies in Ethiopia which reported considerably high prevalence of non-communicable and chronic illnesses [24, 25]. However, the finding in the current study was lower than that of another from Oman; where nearly half of the surveyed households had members with chronic illnesses. Despite this, the specific chronic illnesses reported in the two studies, were comparable. This was evident in that hypertension and diabetes mellitus were reported in high proportions in both studies [13].

The study found that medicine use was high in the area, with nearly half of the households reporting taking medicine during the one month prior to the study. This could be associated with the considerable proportion of households with individuals having chronic illnesses. Another explanation could be the access to various sources of medicines in the town. There are many medicine retail outlets, in addition to the dispensaries in the public and private health institutions. A study done in Porto Alegre (Brazil), with a recall period of 15 days, reported a level of medicine use (54.5%) comparable to the present study [26].

Sharing of medicines was another notable problem identified in the study; it was reportedly practiced in more than one-sixth of the households. This was consistent with a finding in Greece in relation to sharing medicines with relatives, friends and neighbors. However, the Greece study reported a much higher level of sharing within family. The large difference could partly be attributed to the small sample size in the Greece study [27]. The finding on sharing medicines was also lower compared to a finding in Malaysia among female students in a university where more than half reported sharing medicines with friends and families. This could be associated with the difference in the living arrangement where the students lived in a university campus unlike the subjects of the present study [28].

Another study in Ethiopia reported a higher proportion of households engaged in medicine sharing than in this study. The cited study also showed urban areas were less likely to engage in sharing compared to rural areas. The higher figure in the compared study could be attributed partly to the inclusion of rural areas; this might have increased sharing due to lower access to medicine and health services [18]. The habit of leaving medicines unfinished, which was reported by almost all of the households, could indicate an important problem associated with adherence. This could lead to suboptimal therapy and resistance in the case of antibiotics.

The study also found that nearly half of the households had at least one medicine at the time of data collection. This was higher compared to a study done in Tigray region of Ethiopia, and another one from Uganda [29, 30]. However, the finding in this study was fairly comparable to a study done in Nekemte, in the south west of Ethiopia [18]. In contrast, the finding was much lower than results of studies in a number of countries in the Middle East; including United Arab Emirates, Iraq, Iran as well as Oman. According to these findings, medicines were stored in almost all the households studied [11, 13, 31, 32].

In more than half of the households, which reported discarding unused medicines, putting in garbage was the major means of disposal. Discarding in toilets and burning were also reported by a considerable proportion of the households. Similar methods were reported by other studies including in the United Kingdom, Qatar and United Arab Emirates. They reported dumping in garbage of unused medicines as the principal means of disposing, accounting for how two-thirds or more of medicines were disposed [30, 33, 34].

Secondary education, presence of a family member with chronic illness and a health professional in a household were found to predict medicine use during the previous 1 month. In this respect studies which are comparable to the present study were not found. Higher educational level as well as a health professional in a household may have had increased medicine use due to their higher chance of knowing more about medicines and recommending them for various conditions. Chronic illnesses oftentimes require patients to take medicines for a long time. Hence, this could increase the likelihood of medicines use in the households where people with such illnesses reside.

### Limitation

This study was restricted to the urban areas of Gondar town, limiting its generalizability to the rural parts of the town.

## Conclusions

A high level of medicine use among households in the town was found in this study. Medicine use problems such as sharing and a very common habit of leaving medicines unfinished were identified. Secondary education, presence of health professionals as well as members with chronic illness were found to predict medicine use among the households in the study. On the basis of these findings, it is recommended that interventions involving medicine use counseling and education at various levels of the health system are needed. This can be done at hospitals, health centers, clinics and medicine retail outlets; as these are common sources of medicines for households.

## Abbreviations

AOR: adjusted odds ratio
COPD: chronic obstructive pulmonary disease
CI: confidence interval
OR: odds ratio
USD: United States Dollars
